# Barrett’s Esophagus and Intestinal Metaplasia

**DOI:** 10.3389/fonc.2021.630837

**Published:** 2021-06-17

**Authors:** Lu Zhang, Binyu Sun, Xi Zhou, QiongQiong Wei, Sicheng Liang, Gang Luo, Tao Li, Muhan Lü

**Affiliations:** ^1^ Department of Gastroenterology, The Affiliated Hospital of Southwest Medical University, Luzhou City, China; ^2^ Department of Endoscope, Public Health Clinical Medical Center of Chengdu, Chengdu City, China; ^3^ Department of Anesthesiology, West China Hospital, Sichuan University, Chengdu City, China

**Keywords:** Barrett’s esophagus (BE), intestinal metaplasia (IM), endoscopy and pathological identification, monitoring, treatment

## Abstract

Intestinal metaplasia refers to the replacement of the differentiated and mature normal mucosal epithelium outside the intestinal tract by the intestinal epithelium. This paper briefly describes the etiology and clinical significance of intestinal metaplasia in Barrett’s esophagus. This article summarizes the impact of intestinal metaplasia on the diagnosis, monitoring, and treatment of Barrett’s esophagus according to different guidelines. We also briefly explore the basis for the endoscopic diagnosis of intestinal metaplasia in Barrett’s esophagus. The identification techniques of goblet cells in Barrett’s esophagus are also elucidated by some scholars. Additionally, we further elaborate on the current treatment methods related to Barrett’s esophagus.

## Introduction

First reported by Norman Barrett, Barrett’s esophagus (BE) can be simply defined as the presence of columnar epithelium in the esophagus (1). Until 1976, Paull et al. (2) classified the presence of columnar epithelium within the esophagus based on histiological subtypes: Gastric fundic type, junctional type, and specialized type with intestinal metaplasia (IM).

IM refers to the replacement of normal mucosal epithelium with the intestinal epithelium outside the intestine, implying the transformation of a well-differentiated and mature tissue into another differentiated mature tissue under abnormal conditions. IM is common in the stomach and esophagus, but it can also occur in the gallbladder, bile ducts, uterus, bladder, pelvis, ureter, and urethra (3–9).

In recent years, there has been much controversy regarding the role of IM in the diagnosis of BE. We hereby summarize the significance of IM and the evidence supporting the fact that the presence of IM is either mandatory or not to establish a diagnosis of BE. IM in BE is often missed, and several other cells can mimic goblet cells. We cover the classification, manifestation and detection rate of IM under different endoscopic techniques, and methods to accurately identify IM in BE based on endoscopic and pathologic findings. Finally, we also discuss the principles regarding the monitoring and management of BE with and without IM.

## The Significance of IM in BE

IM is considered a precancerous lesion and is closely related to the occurrence of cancer. According to the study of Watanabe et al (10), the presence of IM is associated with the degree of severity of the Barrett’s mucosa, regardless of the size of the tumor. Therefore, they speculated that IM might be an epiphenomenon of BE extension rather than a reflection of the tumor’s origin. However, differentiated mature intestinal epithelial cells can acquire additional mutations, then develop into dysplasia and cancer (11). There is also evidence that IM is associated with a higher frequency of cancer-related mutations compared with nongoblet cell metaplasia (12). Similar to the significance of IM in the stomach (13), although there was evidence that tumor cells may not directly originate from goblet cells (11, 14). It has been undoubtedly suggested that the BE’s mucosa is more prone to neoplastic transformation. In the replacement of the normal esophageal stratified squamous epithelium with columnar epithelium, IM is the only type that is clearly prone to malignant transformation (15).

The possible etiologies for BE include the transcommitment of stem cells or progenitor cells, transdifferentiation of differentiated cells, the expansion of residual embryonic cells located at the squamous-columnar junction (SCJ), and the differentiation of circulating bone marrow cells (16–20). It was not until 2017 that Jiang et al. (21) simulated a BE mouse model with real goblet cells. They described a novel transitional columnar epithelium with distinct basal progenitor cells (p63 + KRT5 + KRT7 +) at the SCJ. After the expression of the caudal-type homeobox transcription factor-2 (CDX2), these progenitor cells were differentiated into intestinal-like epithelium, including goblet cells, resulting in BE with IM. Moreover, such epithelium was found to be localized at the subject’s gastroesophageal junction (GEJ). Moreover, Jin et al. (11) proposed the SPEM model of esophageal IM to explain the multiformity of the stem cells involved in esophageal IM. The differentiated cells and stem cells in this model could be transformed into SPEM-like cells (TFF2+ MUC6+); persistent inflammatory injury eventually led to IM, dysplasia, and carcinogenesis of the SPEM.

## Debate on the Diagnosis of BE

Replacement of the distal esophageal squamous epithelium by metaplastic columnar epithelium forms the pathological basis for BE. Whether IM must be present or whether the length of the columnar mucosa needs to be larger than 1 cm are the main controversial points. The American Gastroenterological Association (AGA), American College of Gastroenterology (ACG), and European Society of Gastrointestinal Endoscopy (ESGE) all believe that IM is necessary for the diagnosis of BE, while the British Society of Gastroenterology (BSG), Asia-Pacific Working Group (APWG) and Benign Barrett’s and Cancer Taskforce consensus group (BOB CAT) all reckon that IM is not needed for the diagnosis of BE (15, 22–26). However, BOB CAT also stressed that the existence of IM should be noted when diagnosing BE (23) (**Table 1**).

**Table 1:** Diagnostic requirements for Barrett’s esophagus in various guidelines.

Category	AGA (2011)	BSG (2014)	BOB CAT (2015)	ACG (2016)	ESGE (2017)	APWG (2016)
Endoscopic performance	CLM	CLM	CLM	CLM	CLM	CLM
Columnar epithelial length	Any length	≥1 cm	Any length	≥1 cm	≥1 cm	≥1 cm
SIM	necessary	not necessary	not necessary, should be noted	necessary	not necessary	necessary
Refs	15	22	23	24	25	26

AGA, American Gastroenterological Association; ACG, American College of Gastroenterology; ESGE, European Society of Gastrointestinal Endoscopy; BSG, British Society of Gastroenterology; APWG, Asia-Pacific Working Group; BOB CAT, Benign Barrett’s and Cancer Taskforce consensus group.

The main basis supporting the fact that IM is mandatory for the diagnosis of BE includes the following: a large number of population-based cohort studies have shown that the risk of esophageal adenocarcinoma is much lower in patients with columnar metaplasia without IM compared to those with IM (27). Compared with columnar metaplasia without goblet cells, tumorigenesis is most commonly seen in columnar metaplasia with goblet cells (12, 28). In-depth evaluation of esophageal biopsy specimens before EMR and esophageal resection specimens after EMR found that IM was found in the columnar esophagus of all the patients with adenocarcinoma (29). Furthermore, some studies have demonstrated that it might be unwise to diagnose a disease that has a negative impact on insurance status and quality of life in non-IM-CLE (Columnar metaplasia without IM) patients (30, 31). The use of IM as a prerequisite for the diagnosis of BE was conducive to the adoption of a more cost-effective approach to the care of BE patients (26).

Evidence supporting the fact that IM is not necessary for diagnosing BE includes the following: a single endoscopic examination and a small number of biopsy samples were not sufficient to rule out IM (32, 33). In the study of Jankowski et al. (34), all the patients with BE who did not have IM at the beginning of the study developed IM during follow-up. Related retrospective studies have reported that patients with or without IM had a similar risk of developing cancer (35). Studies carried out on columnar mucosa resected endoscopically in early cancer subjects have confirmed that cancer may also occur in non-IM columnar epithelium (36). There was also evidence that columnar epithelium without goblet cells may contain similar molecular abnormalities to columnar epithelium with goblet cells (28, 37–39). Lavery et al. (40) reconstructed the cloned ancestor of EAC and provided direct genetic evidence for the malignant tendency of metaplastic columnar epithelium without goblet cells. This is the first direct demonstration that the clonal expansion and precancerous progression of BE are not limited to the metaplastic columnar epithelium with goblet cells.

With or without IM, it was uncertain whether columnar mucosa with a length smaller than 1 cm at the GEJ would progress to cancer. Current research has also depicted that the rate of carcinogenesis in this group of patients is significantly lower than in those with BE (Columnar mucosa more than 1 cm). Thus, most guidelines explicitly require that the diagnosis of BE must include a columnar mucosa exceeding 1 cm at the GEJ (22, 24–26) (**Table 2**). Although BOB CAT did not specify that the columnar mucosa at the GEJ must be more than 1 cm to establish a diagnosis of BE. BOB CAT also indicated that an irregular and ≤ 1 cm lesion at the GEJ may be a natural phenomenon (26). Besides, according to a recent prospective multicenter cohort study, patients with BE lesions smaller than 1 cm will not develop high-grade dysplasia (HGD) or esophageal cancer within 5 years of endoscopic examination (41).

**Table 2:** Surveillance and treatment of BE.

Category	Non-neoplastic BE		BE with LGD	BE with HGD	Refs
AGA^#^	absent definite advice		absent definite advice	endoscopic treatment	/
BSG	BE<3cm, IM(-)	repeat^$^	6months*	Endoscopic treatment	22
	BE<3cm, IM(+)	3-5yearSs*			
	BE≥3cm	2-3years*			
BOB	N		6-12months*	Endoscopic treatment	23
ACG^#^	3-5years*		12months* /Endoscopic treatment	Endoscopic treatment	24
APCS	3-5years*		Endoscopic treatment	Endoscopic treatment	25
ESGE^#^	BE <3 cm	5 years*	BE expert center^&^	BE expert center(&)	26
	BE≥3cm and<10cm	3years*			
	BE≥10 cm	BE expert center^&^			

^#^The diagnosis of Barrett’s esophagusBE requires IM. ^$^For patients with Barrett’s oesophagus shorter than 3 cm, without IM or dysplasia, a repeat endoscopy with quadrantic biopsies is recommended to confirm the diagnosis. If repeat endoscopy confirms the absence of IM, discharge from surveillance is encouraged as the risks for endoscopy probably outweigh the benefits. ^*^surveillance interval. ^N^We make no recommendations about surveillance for nondysplastic BE, but, if undertaken, surveillance should be directed at highrisk groups. ^&^All patients with a BE≥10 cm, a confirmed diagnosis of low grade dysplasia, high grade dysplasia (HGD), or early cancer should be referred to a BE expert center for surveillance and/or treatment.

AGA, American Gastroenterological Association; ACG, American College of Gastroenterology; ESGE, European Society of Gastrointestinal Endoscopy; BSG, British Society of Gastroenterology; BOB, Benign Barrett’s; APCS, Asia-Pacific consensus

In Japan, the 1 cm criteria regarding the morphological changes of BE’s mucous membrane is not mandatory, and such lesions are called ultra-short-segment BE (USSBE). The true meaning of cardiac cancer refers to cancer that occurs in the intestinal metaplastic area of the anatomical cardia or esophagogastric junction (42). By definition, the anatomical position of the cardia and USSBE basically coincided. IM can occur in the cardia and USSBE, and the cardiac mucosa with IM is considered a precancerous lesion of cardiac carcinoma. Over the past few years, with the increasing incidence of cardiac cancer, more attention has been paid to USSBE. Nonetheless, due to the above reasons, the concept of USSBE is not widely used and accepted. In a large sample study in 2016, the annual cancer rate of USSBE was only 0.01% (compared with 0.22% and 0.03% for the long and short segments of BE, respectively) (43). A related etiological investigation also pointed out that BE lesion’s length exceeding 1 cm was not the defining factor (44). However, IM is not required in the definition of USSBE in these studies. If we investigate USSBE with IM exclusively, there may be new results. Monitoring may be crucial for these patients’ prognosis, albeit further research is required to confirm this assumption. Given the high incidence of USSBE in the population, this issue should be taken into account when calculating and comparing the incidence of BE.

## Endoscopic Detection of IM in BE

**Figure 1:**
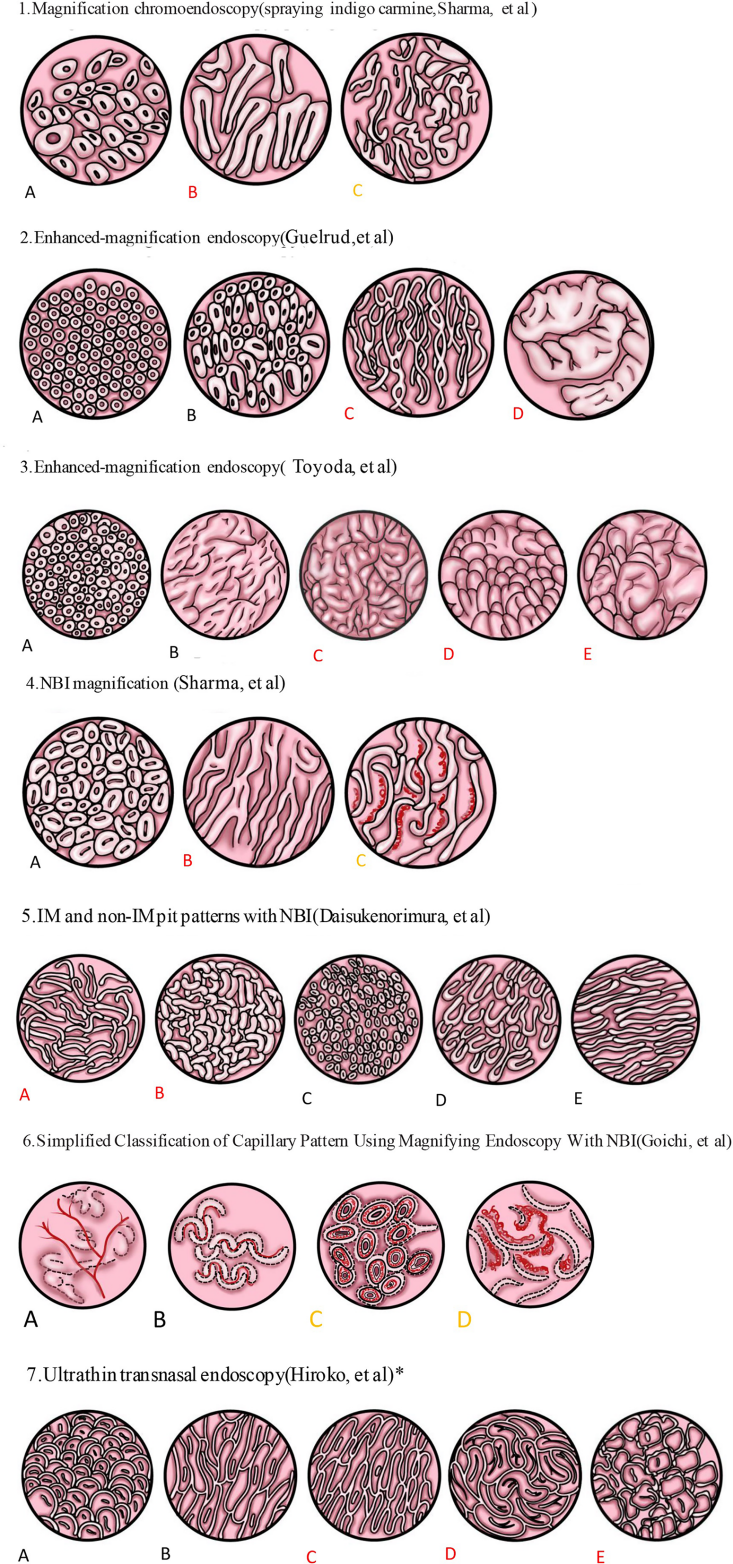
Spraying indigo carmine. **(A)** Circular pattern: a regularly arranged circular or oval area. **(B)** Ridge/villous pattern: a regular arrangement of tortuous and thick villi, such as sausages or cerebriform. **(C)** Irregular/distorted pattern: the villous pattern was obviously distorted and irregular. 2. Enhanced magnifying endoscopy. **(A)** Pattern I: A regularly arranged pattern of circular dots with round pits. **(B)** Pattern II: Circular or oval pits of regular shape and arrangement. **(C)** Pattern III: A regular arrangement of fine villiform appearance with no pits. **(D)** Pattern IV: The thick villi were curled into cerebriform but arranged regularly. 3. Another classification by enhanced magnifying endoscopy. **(A)** Small round pits of uniform size and shape (type 1, corpus). **(B)** Slit and reticular pattern (type 2, cardiac). **(C)** Gyrus pattern (type 3, IM). **(D)** Villous pattern (type 3, IM). **(E)** Mixed gyrus and villous pattern (type 3, IM). 4. NBI images. **(A)** Circular pattern. **(B)** Ridge/villous pattern. **(C)** Irregular and distorted pattern. 5. Another classification by NBI. The pit pattern of BE could be divided into IM and non-IM pit patterns. IM pit patterns included two subtypes: **(A)** tubular, and **(B)** villous types. Non-IM pit patterns included three subtypes: **(C)** round, **(D)** oval, and **(E)** straight types. 6. Simplified classification of capillary pattern in BE by ME-NBI. The capillary pattern was divided into two types: type I: **(A, B)** a branched or rattan-shaped pattern that was clearly shaped and could be tracked smoothly, and type II: **(C, D)** a curly or spiral pattern whose shape was disordered and could not be fully tracked. All dysplasia regions were type II, but there was no significant difference in intestinal phenotype between type I and type II. 7. Pit pattern of SSBE was divided into closed and open types by ultrathin transnasal endoscopy with ME-NBI. Closed pit patterns included two subtypes: **(A)** oval or round pattern, and **(B)** long straight pattern. Open-pit patterns included three subtypes: **(C)** villous pattern; **(D)** cerebriform pattern; and **(E)** irregular pattern. IM was more common in the open type. Black indicated no IM or dysplasia. Red indicated that IM is more likely to be found. Yellow indicated that dysplasia or cancer was more likely to be detected. *indicated an observation of SSBE.

Conventional endoscopy has not been satisfactory in the diagnosis of BE, especially in the detection of precancerous BE lesions. Biopsy of BE lesions is routinely performed from four quadrants superior to the GEJ at intervals of 1-2 cm. According to previous studies, only 10–79% of doctors take biopsy specimens according to this protocol. The higher the length of BE lesions, the lower the application rate of the four-quadrant biopsy principle (45–47). Also, obtaining multiple biopsy specimens and pathological interpretation both imply a significant financial burden (48). BE with IM is a precancerous lesion, which has more significant clinical significance (49). Relevant investigations conducted in Japan suggested that similar to the most common site of occurrence of esophageal adenocarcinoma (EAC), IM often appeared in the 0–3 o’clock area (50). This finding indicates that this area should be the focus of attention and that biopsy specimens taken from this area may increase the detection rate of IM. With the advancement of endoscopic techniques, there are more means for endoscopic detection of IM (**Table 3**, **Figure 1**).

**Table 3:** Endoscopic examination of IM.

Study	Sample Size	Method	Microstructure typing	IM type (sensitivity, specificity)
51	80	indigo carmine+ME	ridged/villous	ridged/villous (97%, 76%)
			circular	
			irregular/distorted	
52	49	acetic acid+ME	I, round pits	III (87%, N)
			II, reticular	
			III, villous	IV (100%, N)
			IV, ridged	
53	95	acetic acid+ME	“corpus” type	“IM” type (85.5%, 92.2%)
			“cardia” type	
			gyrus, villous, or mixed gyrus-villous patterns (“IM” type)	
54	516	acetic acid+ME	different	different (96%, 69%)
55	51	ME-NBI	ridge/villous	ridge/villous (93.5%, 86.7%)
			circular	
			irregular/distorted	
56	54	ME-NBI	IM pit patterns: tubular and villous type	IM pit patterns (92%,97%)
			non-IM pit patterns: round, oval and straight type	
			LBC	LBC (79%, 97%)
57	502	ME-NBI	different	Different (90%, 85%)

NF, Lack of relevant data.

IM, intestinal metaplasia; ME, Magnifying endoscope; ME-NBI, Narrow band imaging magnification procedure; LBC, light blue crests.

### Chromoendoscopy

Methylene blue is a dye that can be absorbed by IM cells giving them a blue coloration, whereas the normal esophageal mucosa does not stain with methylene blue. Studies have shown that methylene blue can improve the detection rate of IM, but subsequent multivariate analysis showed that, compared with the traditional four-quadrant biopsy, the methylene blue stain had no obvious advantage in the diagnosis of BE with IM, BE with dysplasia, and even early cancer. Methylene blue also has a carcinogenic effect (48, 49, 58, 59). Three types of mucosal patterns were noted by Sharma et al. (51) within the columnar mucosa after spraying mucicarmine and using high magnification endoscopy: eidged/villous pattern, circular pattern, and irregular/distorted pattern. The sensitivity, specificity, and positive predictive value of the ridge/villous pattern for detecting IM were 97%, 76%, and 92%, respectively. The sensitivity (100% vs 92%) and specificity (100% vs 69%) of long-segment BE were better than those of short-segment BE. However, there is a lack of sufficient research data.

### Enhanced-Magnification Endoscopy (EME)

EME is a diagnostic method combining acetic acid with magnifying endoscopy. Guelrud (52) first used EME to study the relationship between various histological types and fine structures of mucous membranes and divided the mucosal image into four types: I, round pits; II, reticular; III, villous; and IV, ridged. They found that the IM corresponding to round pits, reticular, villous, and ridged types were 0%, 11%, 87%, and 100%, respectively. In 2003, Toyoda et al. (53) found that pattern III included both villous and slit like patterns, and a new classification was devised as follows: type 1, small round pits of uniform size and shape (“corpus” type); type 2, slit reticular pattern with horizontally elongated mucosal pits (“cardia” type); and type 3, gyrus, villous, or mixed gyrus-villous patterns (“IM” type). The sensitivity, specificity, and diagnostic accuracy of type 3 in predicting IM were 85.5%, 92.2%, and 90.0%, respectively. Even though the specific mucosal classification is different, a large number of studies have demonstrated that EME can accurately identify IM (52, 53, 60). Eight prospective studies in a recent meta-analysis provided data for the diagnosis of IM (54). The combined sensitivity, specificity, positive likelihood ratio (LR), and negative LR for IM were 96%, 69%, 3.0, and 0.06, respectively. This finding suggested that EME is an adequate screening method for IM, and histological confirmation remains key considering its low specificity.

### Narrow-Band Imaging Magnification Procedure (ME-NBI)

ME-NBI allows for detailed examination of mucosal morphology without the use of stains (61). ME-NBI is widely used to diagnose and monitor BE, not only by expert endoscopists but also by non-experts (62). Sharma et al. (55) used ME-NBI to observe 51 patients with BE and divided the BE mucosa into ridge/villous, circular, and irregular/distorted patterns. The sensitivity, specificity, and positive predictive value of the ridge/villous pattern for the diagnosis of IM without HGD were 93.5%, 86.7%, and 94.7%, respectively. Norimura et al. (56) classified pits in BE into IM and non-IM pit patterns. The IM pit pattern comprised two subtypes: Tubular and villous types. Meanwhile, the non-IM pit patterns consisted of three subtypes: round, oval, and straight types. Through ME-NBI, the sensitivity, specificity, positive predictive value, negative predictive value, and accuracy of the IM pit pattern for the diagnosis of IM were 92%, 77%, 76%, 92%, and 83%, respectively. The study also delineated that the appearance of light blue crests (LBC) can be an accurate sign to predict IM in BE. The sensitivity, specificity, and accuracy of LBC for IM were 79%, 97%, and 89%, respectively. Electron microscopy revealed brush borders on the metaplastic epithelium extracted from LBC-positive BE lesions. The appearance of LBC in BE may be closely related to the CD10 antigen present at the brush border, which is also expressed within the LBC-positive mucosa. Researchers speculate that the LBC’s morphology can be explained by the difference in the reflectance of the light at the surface of the ciliated tissue structure (63). Hence, if LBC was detected on NBI, we would always find evidence of IM on histology. However, if LBC was not found on NBI, IM could not be excluded. A meta-analysis (57) elucidated that the targeted biopsy performed by NBI had high sensitivity and specificity for IM and HGD per patient and each lesion. Targeted biopsies by NBI have the same IM detection rate as high-definition white light endoscopy under the Seattle protocol, but require fewer biopsies (64). NBI targeted biopsy could detect more atypical areas of hyperplasia, and the normal surface pattern did not include HGD or EAC. Based on this fact, a biopsy could be avoided in these normal areas. Similar results were obtained from the study conducted by Pascarenco et al. (65), which found that the villus type (According to Shama et al.) had higher sensitivity and specificity for the diagnosis of IM.

To establish a simplified classification, Goichi Uno et al. (61) proposed a new capillary pattern (CP) classification based on the shape and microvessel occupied areas. They divided CP into the following two categories: type I, uniform branched or vine-like pattern with a clear shape that is able to be traced smoothly, and type II, coiled or spiral pattern with a nonuniform shape that cannot be traced sufficiently and with increased vascularity. In this study, all the regions of dysplasia were type II, but there was no significant difference in the intestinal phenotype between type I and type II.

The combination of magnifying endoscopy and NBI makes it easier to detect IM. Although the detection rate of IM has not been greatly improved compared with random biopsy under white light, it has greatly reduced the number of biopsies, which also reduces the number of specimens needed for pathological observation, generating a certain financial benefit. Compared to chromoendoscopy, NBI does not require additional staining, and many large-capacity endoscopy centers use it for routine esophageal observations at no additional cost. This finding indicates its application prospects in the diagnosis and monitoring of BE, but it is still necessary to establish a uniform and reliable standard.

### Linked Color Imaging (LCI) and Blue Laser Imaging (BLI) Systems

In recent years, numerous reports have been published on the application of LCI and BLI in gastrointestinal examinations. Despite the fact that some medical centers have used it to observe the esophagus, there are very few reports on BE. These studies concluded that compared with WLI, LCI improved the visibility of short-segment BE lesions (Esophageal columnar epithelium was less than 3 cm, more than 1 cm, and did not require IM), especially for trainees (66). BLI was deemed extremely helpful for the early detection of synchronous adenocarcinoma in BE patients (67). Reports on the observation of IM in BE with LCI and BLI have not been retrieved. Upper gastrointestinal examination entails continuous observation. Moreover, LCI and BLI have obvious advantages over WLI with respect to the detection of lesions within the stomach To optimize the system for complete detection of lesions in the upper digestive tract, the detection rate for esophageal lesions requires more clinical data.

### Multiphoton Microscopy

Multiphoton microscopy (MPM) based on 2-photon excitation fluorescence and second-harmonic generation allows for simultaneous visualization of cellular details and extracellular matrix components of fresh, unfixed, and unstained tissue (68). MPM can easily identify several cells in the GEJ. For this purpose, the mucosa was classified into a squamous type, columnar stomach type, and metaplastic columnar intestinal-type/BE based on the type of cells identified. Chen et al. (68) showed that goblet cells were identified in 10 out of 25 patients examined by MPM, of which only 7 were diagnosed pathologically as IM. Of the 35 biopsy specimens, 3 (9%) delineated clear goblet cells by MPM observation, which could not be seen in histopathological sections. This study showed that MPM can accurately identify goblet cells, opening a new door for the identification of IM and diagnosis of BE, but its diagnostic efficiency in combination with endoscopy and clinical application still necessitates more exploration.

### Confocal Laser Endoscopy (CLE)

CLE is a combination of traditional video endoscopy and confocal laser microscopy. The addition of confocal laser microscopy enabled us to dynamically identify different cell structures in real-time and make histological observations *in vivo*. In 2006, Kiesslich et al. (69) divided the distal esophagus into three types according to the appearance of different vessels and cell structures: Gastric-type epithelium, Barrett’s epithelium, and neoplastic changes. The predictive sensitivities of BE (with IM) and related tumors were 98.1% and 92.9%, respectively, whereas the specificities were 94.1% and 98.4%, respectively. Studies performed by Richardson et al. (70) also revealed that significantly more IM patients were detected by early CLE users compared with the conventional Seattle protocol. Regarding the detection of BE-related neoplasia, both a clinical randomized controlled trial and meta-analysis showed that CLE combined with targeted biopsy was superior to high-definition white light endoscopy combined with four-quadrant biopsy (71, 72). A recent meta-analysis concluded that CLE is superior to NBI in the detection rate of single lesions (73). However, due to the need to inject a fluorescent agent, the high cost of equipment, and the need for additional learning, the clinical application of CLE is still limited.

### Ultrathin Transnasal Endoscopy

Sugimoto et al. (74) believed that, although present endoscopies used high vision or magnified endoscopy, these endoscopes were of larger caliber and needed sedation. Thus, these endoscopes were not suitable for endoscopic examination. Consequently, they used a newly developed ultrathin transnasal endoscope to closely observe the mucosal structure and investigated the incidence of BE and the usefulness of mucosal structural pattern classification. The study suggested that the new ultrathin transnasal endoscopy is a useful technique for monitoring BE, especially SSBE. The pit pattern was divided into closed (Oval/round and long straight patterns) and open (Villous, cerebriform, and irregular patterns) types. Their results revealed that IM was more prevalent in the open type.

### Computer-Aided Identification System

With the emergence of artificial intelligence, the use of computer-aided identification has increased in the field of endoscopy. The use of artificial intelligence and machine learning techniques have become major aids to cope with pattern prognosis associated with BE, and there have been significantly more research projects on automatic stage classification of BE using different endoscopic techniques (75). Recently, Ghatwary et al. (76) proposed an automatic classification method for the stage classification of BE, which focused on improving the classification of IM. This classification method divides the mucous tissue into four types: Normal scaly tissue, gastric metaplastic tissue, IM, and tumor. This method’s sensitivity and specificity for IM detection were as high as 0.97 and 0.96, respectively. The sensitivity and specificity for the diagnosis of dysplasia and tumors were also over 90%, and the overall accuracy was 96.05%. These aided systems can be used as a second opinion to assist physicians in the diagnosis, as well as training beginners to improve the detection rate of IM. Withal, it should be noted that a huge image data set must be constructed in order to enhance the computer-aided identification system’s overall diagnostic accuracy.

## Identification of Goblet Cells in the Pathological Diagnosis of IM of BE

The presence of goblet cells in the CLM is the pathological diagnosis of IM in BE. However, some non-goblet columnar epithelial cells can mimic goblet cells and impair the diagnosis of IM. Panarelli et al. (77)delineated the characteristics of these cells and their differences from goblet cells. Damaged foveolar epithelial cells containing a copious amount of cytoplasmic mucin can mimic the globular structure of goblet cells and are therefore known as “pseudo-goblet cells,” which can be distinguished from goblet cells due to their pink cytoplasm. A foveal cell that produces enough acid mucin to cause the blue discoloration of the cytoplasm can mimic the blue tone of goblet cells and become a “columnar blue cell.” These cells are cylindrical and can be distinguished from goblet cells due to their difference in shape. The multilayered epithelium is composed of immature-appearing squamoid cells and superficial clusters of columnar cells containing acid mucin. The multilayered epithelium can also simulate goblet cells. Furthermore, all three cell types simulating goblet cells continually fill the surface epithelium diffusely. In contrast, goblet cells are typically individually dispersed in the foveal epithelium background, which is a distinctive feature.

Immunohistochemical staining is commonplace nowadays. Alkaline blue, high iron diamine, Alcian blue, periodic acid Schiff (PAS), CDX2, MUC 2, Heppar1, etc. can all stain goblet cells. However, numerous studies have shown that these types of stains can also stain non-goblet cells, entailing a decrease in specificity (78–86). Although the expression of some immuno-labels in non-goblet columnar epithelium may suggest that IM is more likely to be found elsewhere, it is still not possible to identify goblet cells and non-goblet cells. As a result, the relationship between the staining characteristics of non-goblet epithelium and the risk of dysplasia and/or cancer remains unclear as of yet (78).

Besides, Maskacci et al. (87) demonstrated the combined use of descriptive endoscopy, the suspected esophageal metaplasia range of the Prague standard, and diagnostic charts all conveniently improved the consistency of the CLE interpretation of the esophageal biopsy, which resulted in improved consistency in the diagnosis of IM.

## The Monitoring and Treatment of BE With or Without IM

Although there is a difference in the need for IM in BE diagnosis, only some of the guidelines are specified in the relationship between BE monitoring and treatment strategies and IM (**Table 2**). To optimize the detection rate of IM, ACG (24) suggested that patients who were suspected of having BE (but lacked IM) should undergo a repeat endoscopy within 1 to 2 years, patients with suspected BE should have at least eight random biopsies, patients having short (1–2 cm) segments of suspected BE in which eight biopsies may be unfeasible should have at least four biopsies per cm of circumferential BE, and one biopsy per cm in tongues with BE. BSG (22) suggested that surveillance should consider the presence of IM and the length of BE. For BE, without IM or dysplasia and BE shorter than 3 cm, repeated endoscopic biopsies are recommended to confirm the diagnosis, and if no IM is confirmed repeatedly, de-surveillance is indicated. For patients with BE shorter than 3 cm and accompanied with IM, endoscopic monitoring should be performed every 3–5 years. For patients with BE ≥ 3 cm, endoscopy should be performed every 2–3 years.

The treatment of BE mainly includes drug therapy, anti-reflux surgery, esophagectomy, endoscopic treatment, etc. The main advantage of drug treatment and anti-reflux surgery is that they can alleviate the symptoms of reflux. For simple BE, with or without IM, drug therapy is recommended (23, 24), mainly to bring reflux symptoms under control. If the reflux symptoms are severe and the curative effect of drugs is lacking, anti-reflux surgery can also be considered. Routine endoscopic treatment of BE without dysplasia is not recommended unless the length of BE exceeds 10 cm (26) or is accompanied by nodules (24).

Some breakthroughs in the drug therapy of BE have been made. Initial studies suggest that proton pump inhibitors (PPIs) have a protective effect on the malignant progression of BE (87–89). However, a case-control study accompanied with the follow-up of 9883 patients with BE for up to 10 years showed that the use of PPIs increased the risk of developing EAC or HGD (90). But, a recent large-scale randomized controlled trial of patients with BE showed that high doses of PPIs protect against a composite endpoint of all-cause mortality, EAC, and HGD (34). The study also showed a coupled treatment of PPIs and Aspirin was more effective than PPIs alone, and high doses of PPI (40 mg twice daily) combined with aspirin were the best combination. Aspirin and nonsteroidal anti-inflammatory drugs (NSAIDs) have been reported to reduce the risk of malignant progression of BE, and studies have shown that aspirin is more effective than nonsteroidal anti-inflammatory drugs in reducing the risk of malignant progression of BE (69). Recently, Huo et al. (91) provided a theoretical basis for this. Their work showed that aspirin exerts a unique anti-BE protective effect by acting on IKKβ, inhibiting the action of the nuclear factor-k-gene binding (NF-kB) pathway and inhibiting the expression of CDX2. Also, some auxiliary drugs that are often used in the treatment of gastroesophageal reflux disease (GERD), including medicines that promote gastric motility, neutralize bile acids, and inhibit transient relaxation of the lower esophageal sphincter, which reduces the progression of GERD to BE at varying degrees (92–96) (**Table 4**).

**Table 4:** Drug treatment of BE.

	Mechanism	Curative effect	Refs
PPI	Inhibit gastric acid secretion	improve reflux symptoms, protective effect on the malignant progression of BE	34
aspirin	Inhibits NF-kB pathway activation and CDX2 expression	protective effect on the malignant progression of BE	90
Itobilli	Promote gastrointestinal motility	indirectly improve reflux symptoms, protective effect on the malignant progression of BE	91
Sucralfate	Neutralizes Bile Stomach Acid	Protect the gastroesophageal mucosa	91
Baclofen	Inhibit the excitation of vagus nerve and reduce the occurrence of TLESR	Significantly reduce TLESR, inhibition of acid and non-acid reflux	92, 93
Ursodeoxycholic acid	Increase the expression of antioxidants to prevent DNA damage and NF- kB activation induced by bile acid	protective effect on the malignant progression of BE	94, 95

BE, barrett esophagus; PPI, proton pump inhibitor; NF-kB, nuclear factor-k-gene binding;CDX2, Caudal-type Homebox Transcription Factor-2; TLESR, transit low esophageal sphincter relaxation.

Surgery is still the primary method employed in anti-reflux treatment. Furthermore, laparoscopy gave rise to numerous surgical techniques. The surgical methods mainly include gastric fundus folding, magnetic sphincter augmentation (MSA), electrical stimulation of the lower esophageal sphincter (LES), and bariatric surgery. These procedures all carry merits and shortcomings, but the difficulty of the operation and related complications remain a matter of concern (97–104) (**Table 5**).

**Table 5:** Surgical methods of anti-reflux therapy.

Surgical methods	Anti-gastroesophageal reflux mechanism	advantage	disadvantage	Refs
LNF	The anatomical structure of the esophagus and gastric fundus is completely transformed in the abdominal cavity, and the gastric fundus is folded to form a new anti-reflux barrier	Compared with TIF or PPIs, LNF has a better performance in increasing lower esophageal sphincter stress and reducing esophageal acid exposure time.	Difficult surgery and high incidence of complications (15% -20%)	92, 93
MSA	A series of titanium ring-plated magnets were implanted into the esophagus and gastric fundus junction under laparoscope to increase the tension of the esophageal sphincter and prevent reflux, while retaining the physiological function of the esophageal sphincter.	Significantly improves reflux symptoms, reduces PPI use, and retains snoring and vomiting functions	3.4% to 7% of patients need to remove MSA due to complications. Whether magnets can remain in the body for a long time needs further evaluation.	94, 95
LES	The electrodes and pulse transmitters are implanted into the lower esophageal sphincter under the laparoscope, and the frequency and intensity of the stimulus are adjusted by an external editor to control the lower esophageal sphincter contraction	Significantly improved symptoms and acid exposure time, PPI can be discontinued in all patients, and side effects associated with treatment are low	Large sample clinical studies are currently lacking	96, 97
bariatric surgery	High visceral pressure in obese patients will lead to increased intragastric pressure, forming a pressure gradient conducive to reflux, and more prone to hiatal hernia	At the same time, weight loss can be improved, and patients’ symptoms have improved. Most patients can stop using PPI.	22% of GERD patients still have symptoms Persistent; Suitable only for obese patients; Destroyed physiological structure	98, 99

LNF, laproscopic Nissen fundoplication; MSA, magnetic sphincter augmentation; LES, electrical stimulation on the lower esophageal sphincter; TIF, transoral incisionless fundoplication; PPI, proton pump inhibitor; GERD, Gastroesophageal Reflux Disease.

The field of endoscopy is constantly evolving. Examples of endoscopic anti-reflux surgery include trans-oral incision-free fundoplication (TIF), radiofrequency ablation (Stretta), anti-reflux mucosectomy (ARMS), and endoscopic injection or implantation (105–112) (**Table 6**).

**Table 6:** Endoscopic methods for anti-reflux therapy.

Surgical methods	Anti-gastroesophageal reflux mechanism	advantage	disadvantage	Refs
TIF	Re-establishment of esophageal and gastric fundus junction with stapler	Good anti- reflux effect, effectively improve symptoms(TIF vs PPIs, 67% vs 45%, P=0.023)	Poor long-term efficacy, the effect of esophageal and gastric fundus folding gradually diminishes over time	100–102
Stretta	Use of RF current at the junction of the esophagus and gastric fundus to form local scars and fibrosis, reduce tissue compliance and inactivate part of the nerves of the lower esophageal sphincter, thicken LES and increase tension, thereby reducing the frequency of transient LES relaxation	Simple operation, fewer complications, and improved reflux symptoms; Studies have shown that about 42% of patients can stop PPI during long-term follow-up	Studies have shown that it is equivalent to the sham treatment group, and a larger sample of randomized clinical trials is needed to prove its efficacy	103, 104
ARMS	Apply EMR or ESD to remove the semicircle or 2/3 or more of the mucosa at the junction of the esophagus and gastric fundus to form a relatively narrow and resist reflux	Significantly improved symptoms, good anti-reflux effect, PPI can be discontinued in all patients	The technical difficulty is relatively greater, and the current data are mostly small sample studies	105
RAP	Half-peripheral gastric mucosal resection and full-layer folding of lower esophageal sphincter and cardiac	Combining the advantages of ARMS and TIF to effectively improve symptoms, most patients can get rid of PPI dependence (8/10)	Exploration phase, small sample study	106
endoscopic injection or implantation	Injecting or implanting an inert material into the esophagogastric gastric junction causes the tissue to swell and form an anatomical reflux barrier	Theoretically feasible	The efficacy and safety are poor, and there is a risk of damaging adjacent structures such as the aorta, which needs to be further explored	107

TIF, transoral incisionless fundoplication; Stretta, Stretta radiofrequency ablation; ARMS, anti-reflux mucosectomy; RAP, resection and plication; PPI, proton pump inhibitor; LES, lower esophageal sphincter; EMR, Endoscopic Mucosal Resection; ESD, Endoscopic Submucosal Dissection

Generally, surgery or endoscopic treatment is only indicated in BE that is more than 10 cm in length or is accompanied by nodules, intraepithelial neoplasia, or cancer (24, 26). Esophagectomy can achieve complete eradication, but the quality of life is seriously impaired, and its mortality rate is high. There are many kinds of endoscopic therapy, including endoscopic mucosal resection (EMR), endoscopic submucosal dissection (ESD), radiofrequency ablation (RFA), argon plasma coagulation (APC), contact cryo-balloon focal ablation system (CbFAS), and so on (**Table 7**). Compared with other methods, endoscopic treatment possesses the merits of high safety, conserving the normal function of the esophagus and eradicating lesions simultaneously.

**Table 7:** Comparison of endoscopic treatments for different lesions surrounding BE.

Surgical methods		f-EMR+ RFA vs EMR	EMR vs ESD	ESD	RFA	CbFAS
Number of cases included		774 vs 751	20 vs 20	524	136	41
research method		systematic review and pooled-analysis	Retrospective study	meta-analysis	Retrospective study	Prospective study
Lesion type		BORN	HGIN/EAC	HGIN/EAC	BORN	ImAC/HGD/LGD
Follow-up time		12 mouth	23.1+/-6.4 mouth	22.9 mouth	27.5 mouth	
CE-N		93.4% vs 94.9%	93.8% vs 94.1%	/	98.5%	95.0%
CE-IM		73.1% vs 79.6%	37.5% vs 58.8%	/	77.9%	88.0%
R0 Resection rate		/	11.8% vs 58.8%	74.5%	/	/
Recurrent neoplasia rate		1.40%	0% vs 5.0%	0.16%^*^	4.5%	/
Dysplasia recurrence rate		2.60%	/	/	/	/
recurrent IM rate		16.10%	/	/	15.0%	
Serious complications	stricture	10.2% vs 33.5%	/	11.6%	/	9.7%
	bleeding	1.1% vs 7.5%	/	1.7%	/	2.4%
	perforation	0.2% vs 1.3%	0% / 10.0%	1.5%	/	0.0%
Refs		113	114	115	116	117

f-EMR, Focal Endoscopic Mucosal Resection ; RFA, radiofrequency ablation; ESD, Endoscopic Submucosal Dissection; EMR, Endoscopic Mucosal Resection; CbFAS:

BORN: Barrett’s esophagus (BE) related neoplasia; CE-N, complet cryoballoon focal ablation system; e eradication of neoplasia; CE-IM, complete eradication of intestinal metaplasia; HGIN, high-grade intraepithelial neoplasia; EAC, Esophageal adenocarcinoma; HGD, high-grade dysplasia; LGD, Low-grade dysplasia; endoscopic submucosal dissection; R0, higher rates of complete resection; ImAC, Intramucosal cancer; /: Indicates that there is no corresponding data for this study. ^*^Recurrence of the patients with R0 resection, histology showing well-to-moderate differentiation and no lymphatic invasion.

Patients suffering from esophageal intramucosal cancer who have undergone EMR and esophagectomy had a similar long-term recurrence rate and mortality (118). RFA is a mature endoscopic technique for the eradication of IM with dysplasia (116). EMR and RFA are usually combined in the treatment of early tumors of BE. According to a recent study (113), the focal EMR followed by RFA has a similar efficiency level as EMR but is safer than EMR in patients with HGD/EAC. However, due to the decreased access to specimens, the uncertainty of the damage to the anatomical layer, and high costs, RFA is not commonplace in Asia. For larger lesions, EMR can only be segmented and resected multiple times. The Japanese view is that the single resection of ESD is the preferred choice regardless of the size of the lesion due to the blind resection line in patients suffering from adenocarcinoma (119). However, due to the long-term learning curve and the high rate of adverse events, the use of ESD in the West is still limited. A study has shown that the complete remission rate from neoplasia of ESD is the same as that of EMR at 3 months. ESD can potentially cause severe adverse events (two cases of perforation) because of its long operation time (114). However, according to the results of a multicenter study from the West, ESD is effective and safe and can achieve a good level of proficiency after approximately 30 operations (120). A recent meta-analysis also shows its effectiveness and safety (115).

The remission rate of APC in the treatment of BE ranged from 64.9%-94.7%. Like previous endoscopic treatments, esophageal stricture was still the most commonly observed complication (121). To prevent postoperative esophageal stricture, hybrid APC was developed based on APC, that is, submucosal fluid injection before APC treatment to reduce the depth of coagulation which in turn reduces the incidence of esophageal stricture (122). In vitro animal experiments showed that the coagulation depth of hybrid APC was shallower than that of conventional APC (123). In the esophageal wall level analysis, the two methods were consistent in the injury of the epithelium, lamina propria, and muscularis mucosae. The number of submucosal injury cases by conventional APC was more significant, and only conventional APC damaged the proper muscle layer. In humans, it was found that the common observation remission rate of hybrid APC for BE was 96%, the pathological observation remission rate was 78%, and the postoperative stenosis rate was only at 2% (122).

Recently, CbFAS has been developed for esophageal mucosal ablation, which freezes the target mucosa to -85 degrees Celsius to achieve ablation of the BE mucosa (124). According to a recent prospective trial (117), the overall 1-year complete eradication of all dysplasia (CE-D) and complete eradication of intestinal metaplasia rates of BE patients with neoplasia was 95% and 88%, respectively. However, the CE-D rate of ultra-long BE, with length ≥ 8 cm, was only at 67%. The main complications were esophageal stricture (9.7%) and upper gastrointestinal bleeding without treatment (2.4%). Based on the current data, CbFAS can be used in the primary or emergency treatment of BE-related tumors and IM, but there is a lack of large-scale clinical research data to evaluate this technique.

This review summarizes BE and IM from the aspects of the definition, endoscopic recognition, pathology, diagnosis, and treatment. It is believed that there will be more detection and treatment in the future.

## Author Contributions

LZ and BS drafted this review. XZ and QW assisted in table making and sketching. SL and GL edited the grammar, text format, and framework, etc. TL put forward some essential suggestions on the content of the article. ML provided the direction and ideas of writing and made repeated revisions and guidance in the whole process of writing. All authors contributed to the article and approved the submitted version.

## Funding

This research was supported by the NFSC (No. 81672458 and No. 81972996), Sichuan provincial Science and Technology Department (No. 2017JQ0052), and the Science and Technology Strategic Cooperation Programs of Luzhou Municipal People’s Government and Southwest Medical University (No. 2016LZXNYD-T06). Talent development project of The Affiliated Hospital of Southwest Medical University.

## Conflict of Interest

The authors declare that the research was conducted in the absence of any commercial or financial relationships that could be construed as a potential conflict of interest.

## Abbreviations

BE, Barrett Esophagus; IM, Intestinal Metaplasia; USSBE, ultra-short-segment BE; GERD, gastroesophageal reflux; HGIN: high-grade intraepithelial neoplasia; HGD: high-grade dysplasia; LGD: Low-grade dysplasia; EAC: Esophageal adenocarcinoma; SCJ, squamous-columnar junction; CDX2, caudal-type homeobox transcription factor-2; EME, Enhanced-magnification endoscopy; ME-NBI, Narrow-band imaging magnification procedure; LCI, Linked Color Imaging; BLI, Blue Laser Imaging; MPM, Multiphoton microscopy; CLE, Confocal laser endoscopy; PPI, proton pump inhibitors; TIF, transoral incisionless fundoplication; RAP, resection and plication; ARMS, antireflux mucosectomy; EMR, endoscopic mucosal resection; ESD, endoscopic submucosal dissection; RFA, radiofrequency ablation; APC, argon plasma coagulation; CbFAS, contact cryoballoon focal ablation system.
